# Determination of Translaminar Notch Fracture Toughness for Laminated Composites Using Brazilian Disk Test

**DOI:** 10.3390/polym14163246

**Published:** 2022-08-09

**Authors:** Ali Reza Torabi, Mohammad Amin Motamedi, Bahador Bahrami, Meghdad Noushak, Sergio Cicero, José Alberto Álvarez

**Affiliations:** 1 Fracture Research Laboratory, Faculty of New Sciences and Technologies, University of Tehran, Tehran 14395-1561, Iran; 2 Fatigue and Fracture Research Laboratory, School of Mechanical Engineering, Iran University of Science and Technology, Narmak, Tehran 16846, Iran; 3 LADICIM, Departamento de Ciencia e Ingeniería del Terreno y de los Materiales, Universidad de Cantabria, Avenida de los Castros, 44, 39005 Santander, Spain

**Keywords:** failure of notch, VIMC-MTS, VIMC-MS, laminated composite, VIMC, fracture

## Abstract

This paper evaluates the fracture of notched epoxy matrix composites using the Brazilian disk (BD) test from both numerical and experimental points of view. The study began with a comprehensive experimental program covering three different composite lay-ups (quasi-isotropic, unidirectional, and cross-ply) and various geometries of U and V notches. Specifically, the BD samples combined the three layouts, four different notch angles, and three notch radii with three specimens per combination, leading to an overall number of 108 fracture tests. The experiments showed the appropriateness of the BD test for the study of the fracture behavior of composite materials and provided a good pool of data for further investigations. Subsequently, the virtual isotropic material concept (VIMC) was applied in combination with two fracture criteria to theoretically predict the experimentally acquired fracture loads. This study demonstrated that using the VIMC approach can provide robust predictions while incurring much lower computational costs compared to the conventional approaches found in the literature.

## 1. Introduction

Over the past decades, many theoretical, numerical, and experimental studies have been devoted to the analysis of fracture and stress fields in specimens made of laminated composites and subjected to various loading conditions [1,2]. The majority of them, however, have dealt with cracked samples rather than notched ones. Nevertheless, the existence of notches in composite structures is unavoidable due to the design requirements. These generate various cut-outs and holes in several geometries, such as bean-shaped holes with two U- or V-ends, rectangular cut-outs with round corners, etc., and lead to a stress concentration at their corners, increasing the possibility of damage initiation and final fracture. Due to the broad demand for composites in the aircraft and automotive industries and the wide range of fiber and epoxy properties [3,4] and lay-up configurations, a reliable, user-friendly, and straightforward criterion is needed to ensure the structural integrity and to predict failures in components made of laminated composites.

According to a thorough literature survey, there are three main distinct approaches for the fracture prediction of composite samples: (i) fracture-mechanics-based approaches [5,6,7,8,9], (ii) models based on the stress-fracture [10,11,12,13,14,15,16,17,18,19], and (iii) progressive damage models [20,21,22,23,24,25,26,27,28]. In the fracture-mechanics-based approach, the formation and growth of damage in the stress concentration region is evaluated. Damage in composites can be in the form of delamination, fiber and matrix breakage, fiber debonding, matrix yielding, etc. [7]. For instance, the damage zone model (DZM) proposed by Backlund and Aronsson [7,8] calculates the residual strength of composite laminates. The inherent flaw model (IFM) is one of the well-known failure models based on linear elastic fracture mechanics (LEFM) and requires two main inputs: the strength in un-notched conditions and the length of inherent flaws [6]. In the stress-fracture-based models, the average stress and the point stress (PS) are two common criteria suggested by Whitney and Nuismer [10]. Pipes et al. [18] and Kim et al. [19] tried to modify and improve the predictions of the PS criterion by suggesting a more complex model with a higher number of input parameters. The Mar–Lin criterion [15] is another stress-fracture criterion that states that the failure in multi-layer composites occurs through the crack propagation in the matrix. Finally, progressive damage models have been used to analyze the damage- and stress-distribution variations that result from the damage progress [25,26,27]. These approaches mainly provide an analysis of the resulting stress concentrations. For instance, Tan [22] proposed a progressive-damage finite element (FE) model for in-plane loading conditions, which analyzed the laminated composites element by element based on the corresponding stress concentration. In this model, stress redistributions can be computed using the boundary conditions and the stiffness matrix of the damaged structure. After that, Coats and Harris [27] developed a methodology for predicting the fiber fracture and matrix cracking for different notch sizes by calculating the residual strength.

The nature of fractures in composites is more similar to that in brittle materials than that in ductile ones due to the sudden occurrence of a failure. Despite the difficulty of fracture analysis in anisotropic composite materials, the analysis of fractures in brittle isotropic materials is easier and more straightforward. Various failure theories, such as the strain energy density (SED) [29], the cohesive zone model (CZM) [30,31], the maximum tangential stress (MTS) [32], the mean stress (MS) [33], and the J-integral [34], have been proposed for brittle fracture analysis of isotropic materials. Recently, Torabi and Pirhadi [35] proposed the virtual isotropic material concept (VIMC) for predicting the failure in composites by treating them as an isotropic material. They proved that while the VIMC is fast and easy to apply, it also provides accurate estimations [36].

Although the Brazilian disk (BD) is a well-known test in the fracture toughness evaluation of isotropic brittle materials, there is no significant research history on applications of this test for composite materials. In this paper, the BD test was utilized to perform a comprehensive experimental and analytical study on the fracture of notched composite laminates. Subsequently, using the VIMC combined with the MTS and MS criteria, the translaminar fracture toughness of each configuration was predicted. The robustness and simplicity of the current approach were proven by comparing the analytical and the experimental results.

## 2. Materials and Methods

### 2.1. Material Characterization

To produce the composite laminates, the thermoset epoxy resin, EPON 828, and E-glass fibers were used as the matrix and the reinforcement, respectively. The EPON 828 produced by Hexion (Florida, USA) has strong adhesion properties, making it a suitable choice for creating composite materials. The advantages of the epoxy resin include high strength, low viscosity, low volatility during the curing process, low shrinkage rate, which prevents the increase in shear stresses at the interface between epoxy and fibers, and its incredible variety. The density of EPON 828 in this study was 1189.33 Kg/m^3^. Appropriate shear strength, good electrical insulating and dielectric properties, and compatibility with different curing agents have made this epoxy popular in various applications, such as fiber-reinforced pipes, tanks, composites, electrical encapsulations, etc. A vacuum bag-autoclave molding with bleeders on the top and bottom surfaces was utilized for manufacturing the composite plates, ensuring that the fabrication process is controlled for GFRP (Glass-Fiber-Reinforced Plastic) composites. The molding in the autoclave was performed in three different stages. Initially, specimens were cured at room temperature. Then, they were placed at 60 °C for two hours. After that, the specimens were post-cured for three hours at 120 °C. Three lay-up configurations were manufactured for this study: unidirectional ((0)_s_), cross-ply ((0/90/0/90)_s_), and quasi-isotropic ((0/90/±45)_s_). The samples were fabricated by using a CNC waterjet cutting machine to create a U-notch and V-notch with the required geometries on the specimens.

The fiber volume in the composite plates was in the range of 56% to 59%, and each lamina was around 0.28 mm-thick, generating an overall thickness between 5.6 mm and 5.8 mm for the 16 plies of each laminate configuration. 

**Figure 1:**
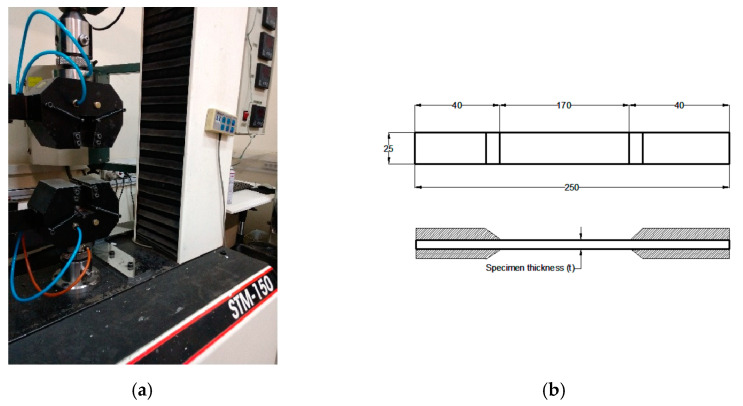
(**a**) The universal testing machine, SANTAM 150, and (**b**) specimen schematic based on the ASTM D3039 [37].

To measure the ultimate tensile strength of the different lay-ups, tensile tests (3 per lay-up configuration) were carried out using a universal tension–compression test machine at the loading rate of 2 mm/min, according to the ASTM D3039 [37], as seen in Figure 1. The tensile specimens were clamped using the pneumatic jaws of the universal testing machine, SANTAM 150. The tension load was then applied at the loading rate of 2 mm/min, according to the ASTM D3039 [37], until the last ply failure of the laminated composite. It is worth noting that the approach utilized in the present study does not need E_x_, E_y_, ν_x_, and ν_y_, which are the elastic moduli and Poisson’s ratios in the X and Y directions; only two parameters were required: the ultimate tensile strength (σ_u_) and the translaminar fracture toughness (K_TL_). Hence, it is not necessary to conduct two separate tensile tests in the X and Y directions, as is typical in most experimental composite research studies.

**Figure 2:**
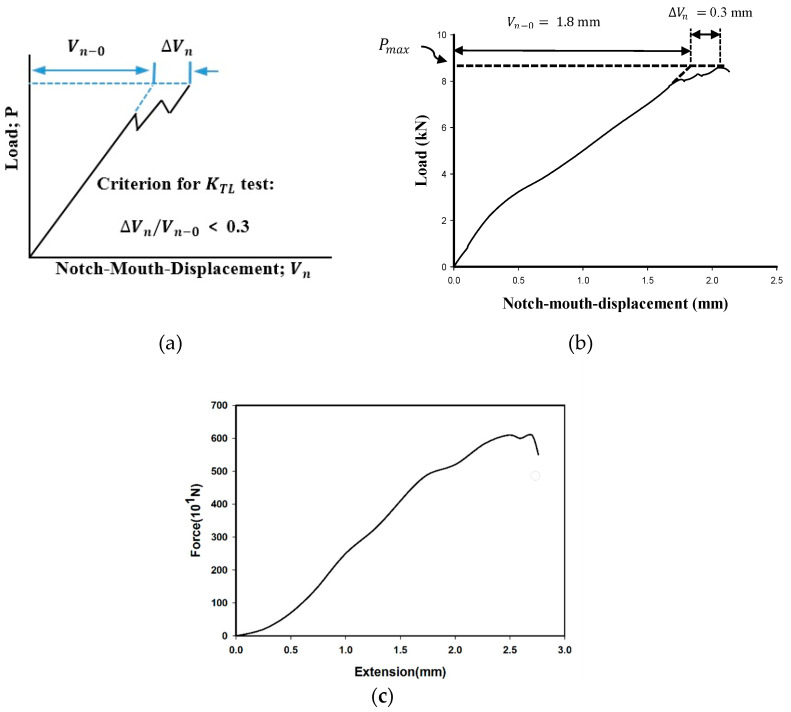
(**a**) Schematic of the load versus the NMD according to the ASTM E1922 [38]; (**b**) a sample load versus the NMD curve corresponding to the quasi-isotropic laminates. (**c**) The load–displacement curve for U-notched cross-ply configuration with a notch radius of 1 mm.

K_TL_ was measured using three fracture tests on the test configuration described in the ASTM E1922 [38]. It should be noted that in order for the test to be valid, the notch-mouth displacement (NMD) value should satisfy the criterion, ∆V_n_/V_n−0_ ≤ 0.3, as depicted in Figure 2a.

After conducting the tests, the criterion stated in the ASTM E1922 [38] was implemented to check the validity of the translaminar fracture toughness tests. As an example, for a quasi-isotropic specimen, V_n−0_ and ∆V_n_ are 1.8 mm and 0.3 mm, respectively. Therefore, ∆V_n_/V_n−0_ equals 0.166, which meets the requirement of being less than 0.3 (see Figure 2b). Then, the highest value recorded from the load–displacement curve in each fracture toughness test was considered for measuring $K_{TL}$. Table 1 lists the mechanical properties obtained for the tested samples. The $K_{TL}$ of a laminate can be ascertained using the ASTM E1922 [38], which indicates the capacity of a laminate for tolerating the propagation of a pre-existing translaminar crack. Based on the VIMC approach, the fracture toughness of the virtual isotropic material was assumed to be equal to the $K_{TL}$ value of a composite laminate. Furthermore, the modulus of elasticity (E) was obtained from the tension test for each lay-up configuration. In other words, E was not measured for each layer separately, but rather measured for the bulk of the composite laminate. 

### 2.2. Fracture Tests on Brazilian Disk Samples 

**Figure 3:**
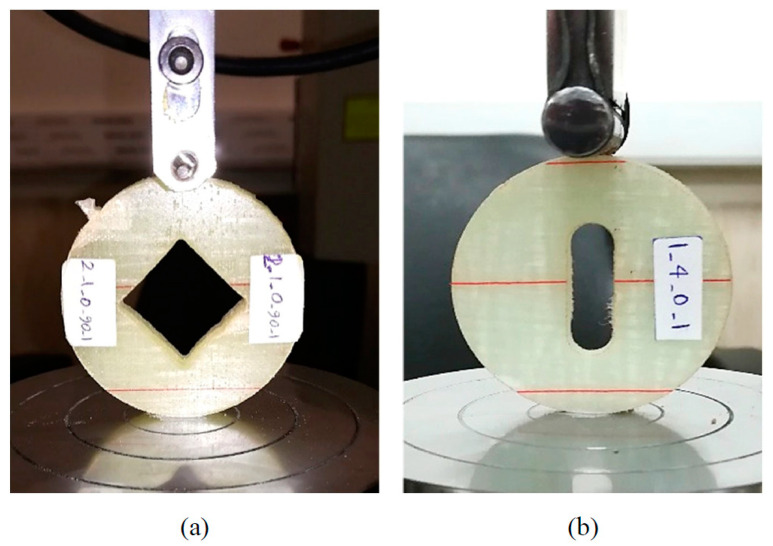
Setup configuration used for conducting Brazilian disk tests; (**a**) V-notched specimen (90°); (**b**) U-notched specimen.

Figure 3 shows the test setup used for conducting the Brazilian disk tests. To manufacture the specimens, a CNC (Computer Numerical Control) water jet machine was employed and, subsequently, the cut surfaces were polished using a brass rod. The following dimensional parameters were considered in the fabrication of the samples: The diameter of the disk (D) was 60 mm and the distance from the bottom notch tip to the top one was half of D. The round-tip V-notch (RV-notch) opening angles (2α) were 30, 60, and 90 degrees. By decreasing the notch opening angle to zero, the RV-notch geometry was converted to a U-notch. Figure 3 shows the compressive test setup and the schematic of the Brazilian disk test specimens. Moreover, three notch-tip radii of 1, 2, and 4 mm were selected for the samples. Three types of composite lay-ups, four notch angles, three notch-tip radii, and three repetitions of each test made up an overall number of 108 mode-I fracture tests.

After conducting the tests, the peak load of each load–displacement curve was considered as the fracture load. The obtained values are reported in Table 2, Table 3 and Table 4. It can be observed that, for all notch geometries, the fracture load increased when increasing the notch-tip radius. This is due to the fact that larger notch-tip radii lead to lower stress concentrations at the notch root.

### 2.3. The Virtual Isotropic Material Concept (VIMC)

The VIMC equates a real laminated composite with orthotropic behavior to a virtual brittle plate of the same geometry with isotropic behavior [35]. In the VIMC, the bulk behavior of the composite specimens is essential, and the basis of the concept is assuming an equivalent brittle isotropic material instead of a composite material. In the VIMC, the microscopic behavior of the material is not important, and the last ply failure as a macroscopic failure is considered.

When dealing with the fracture analysis of engineering materials containing notches, there are two main material parameters: the characteristic strength (σ_f_), which is generally assumed to be equal to the material ultimate tensile strength (σ_u_), and the fracture toughness (K_c_). At the same time, the VIMC requires two important properties of the laminated composite to be defined: the ultimate tensile strength (σ_u_) and the trans-laminar fracture toughness (K_TL_), whose correct definition is the main difficulty of the application of the VIMC. However, once defined, they are considered as the K_c_ and the σ_u_ of the virtual isotropic material, with the fracture assessment of the laminated composites following the same methodologies as those used for isotropic materials. Thus, it is worth noting that the VIMC eliminates the need for time-consuming and costly experiments to determine the longitudinal elastic modulus (E_x_), lateral elastic modulus (E_y_), and shear modulus (G_xy_).

**Figure 4:**
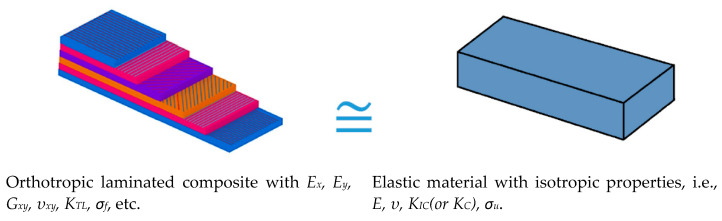
Schematic of the VIMC.

Schematically, the VIMC is depicted in Figure 4. According to the VIMC, a multi-layer material with different behaviors and properties in the layers, such as E_x_, E_y_, G_xy_, ν_xy_, K_TL_, σ_f_, etc., may be treated as an isotropic material with the primary mechanical properties of E, K_IC_, and σ_u_. σ_u_ and K_IC_ are the ultimate tensile strength and the plain strain fracture toughness, which are two essential parameters in all brittle fracture criteria. The MS and MTS criteria are combined with the VIMC below to predict the critical loads of BD specimens.

### 2.4. The MTS and the MS Criteria

**Figure 5:**
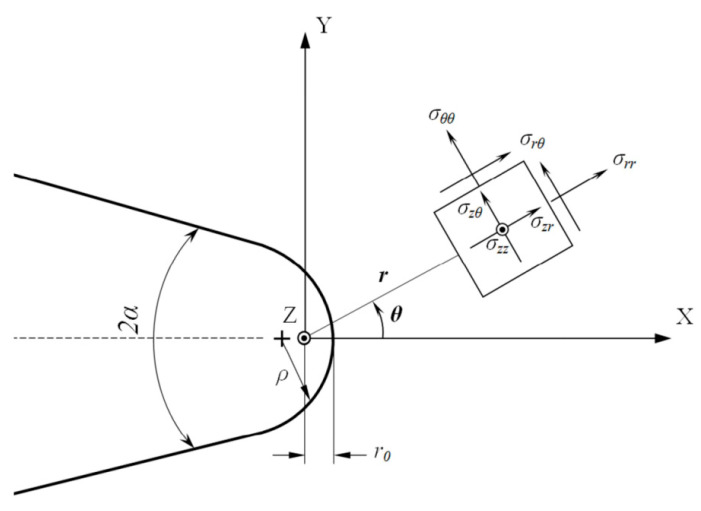
Blunt V-notch with its coordinate system and the resulting geometrical parameters.

The maximum tangential stress (MTS) criterion was proposed by Erdogan and Sih [39] to evaluate the mixed-mode I/II failure of brittle components. According to the MTS approach, brittle fracture occurs if the tangential stress (σ_θθ_, see Figure 5) at the critical distance (r_c_) in front of the notch tip equates to the critical stress, σ_c_. Ritchie et al. [40] presented the equation for r_c_ as follows:

r_c_ = (1/2π)·(K_IC_/σ_c_)^2^ (1)

According to [41], for most brittle materials, σ_c_ may be assumed to be equal to σ_u_.

By modifying the existing relationship for calculating the fracture toughness of U-notched isotropic materials [38], the fracture toughness of U-notched composite specimens in accordance with the VIMC and MTS can be written as:

K_TL_^U,ρ^ = σ_u_ · (π(ρ + 2r_c_))^1/2^/[1 + (ρ/(ρ + 2r_c_))] (2)

where K_TL_^U,ρ^ is the translaminar fracture toughness for a U-notched composite specimen with a notch radius of ρ. Similarly, the following equation can be used for V-notched composite samples [36]:

K_TL_^V,ρ^ = σ_u_ · (2π)^1/2^(r_0_ + r_c_)^(1−λ1)^/[1 + (1 + r_c_/r_0_) · n_θθ_(0)] (3)

where K_TL_^V,ρ^ stands for translaminar fracture toughness for a V-notched composite specimen with the notch radius of ρ; the parameter r_0_ denotes the distance between the polar reference frame origin and the notch tip (see Figure 5), and the function, n_ij_(θ), and the eigenvalue, λ_1_, are related to the notch opening angle.

Concerning the mean stress (MS) criterion, it states that brittle fracture occurs when the average tangential stress over a critical distance (d_c_) in front of the notch tip reaches the critical stress of the material being analyzed (σ_c_) [36]. Seweryn [41] proposed the critical distance of MS criterion as follows:

d_c_ = (2/π) · (K_IC_/σ_c_)^2^ (4)

Using the same argument as that presented for the MTS criterion, the equation for translaminar fracture toughness in U-notched specimens in accordance with the MS criterion can be written as:

K_TL_^U,ρ^ = σ_u_·(2π)^1/2^·d_c_/(2 · d_c_*^1/2^ − ρ/d_c_*^1/2^) (5)

where K_TL_^U,ρ^ is the translaminar fracture toughness of a U-notched composite specimen with the notch radius of ρ. In the above equation, d_c_* follows Equation (6):

d_c_* =d_c_ + ρ/2. (6)

The V-notch translaminar fracture toughness (K_TL_^V,ρ^) is also quantified as:

K_TL_^V,ρ^ = σ_u_·(2π)^1/2^ · d_c_/{(1/λ_1_) · (d_c_*^λ1^ − r_0_^λ1^) + (n_θθ_(0)/μ_1_ · r_0_^μ1 − λ1^)·(d_c_*^μ1^ − r_0_^μ1^)} (7)

where μ_1_ is also an eigenvalue dependent on the notch opening angle. Using Equations (1) and (4) and inputting the values of σ_u_ and K_TL_, as reported in Table 1, both r_c_ and d_c_ (for each lay-up configuration) can be easily calculated.

### 2.5. Finite Element Analysis

**Figure 6:**
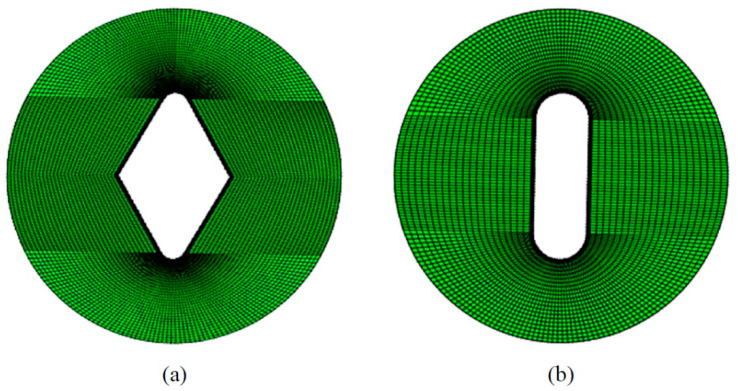
Examples of mesh patterns: (**a**) V-notched specimen (2α = 60°); (**b**) U-notched specimen.

It is mandatory to obtain the stress field around the notches to implement the VIMC approach on different notch samples and, subsequently, predict the fracture load. This paper uses the numerical method of the finite element (FE) to calculate the stress field around different notches. The notched composite specimens were modeled as isotropic solids. Due to the low thickness of the samples (about 5.7 mm) compared to the diameter (60 mm), the thickness-to-diameter ratio was less than 10%, so the simulations were conducted assuming plane-stress conditions. The models were also discretized using eight-node quadratic elements. As seen in Figure 6, because of the high stress gradient at the notch-tip neighborhood, a finer mesh size was employed at this region. Each FE model contained around 120,000 elements in total. 

As can be seen from Equations (2), (3), (5), and (7), the results of the two failure criteria are in the form of the translaminar notch fracture toughness (TLNFT), whereas the experimental results listed in Table 2, Table 3 and Table 4 are given as critical loads. To make the predictions comparable to the corresponding experimental results, the critical loads presented in Table 2, Table 3 and Table 4 should be converted into the associated values of TLNFT. To do so, the critical load of each notched specimen was applied to the associated FE model and, after a linear elastic stress analysis, the maximum tensile stress at the notch tip (σ_max_) was extracted. By substituting this stress value into Equations (8) and (9) for the U-notched and V-notched specimens, respectively, which are the expressions of the notch stress intensity factor (NSIF), the experimental results in terms of the TLNFT were derived. Note that Equations (8) and (9) were taken from [42,43], and they define the equations for obtaining the mode I stress intensity factor for U- and blunt V-notches, respectively. For the sake of brevity, details of deriving these equations are not provided. Moreover, more details necessary to obtain the values of the parameter ω_1_ can be found in [42,43].

K_I_^U, ρ^ = σ_max_ · (πρ)^1/2^/2 (8)

K_I_^V, ρ^ = σ_max_ · (2π)^1/2^ · r_0_^1 − λ1^/(1 + ω_1_) (9)

where K_I_^U, ρ^ and K_I_^V, ρ^ are the U-notch and V-notch stress intensity factors with a notch-tip radius of ρ, respectively. The σ_max_ is the maximum stress at the notch round edge and the λ_1_ is the eigen value that is a function of the opening angle of the notch. 

## 3. Results and Discussion

**Figure 7:**
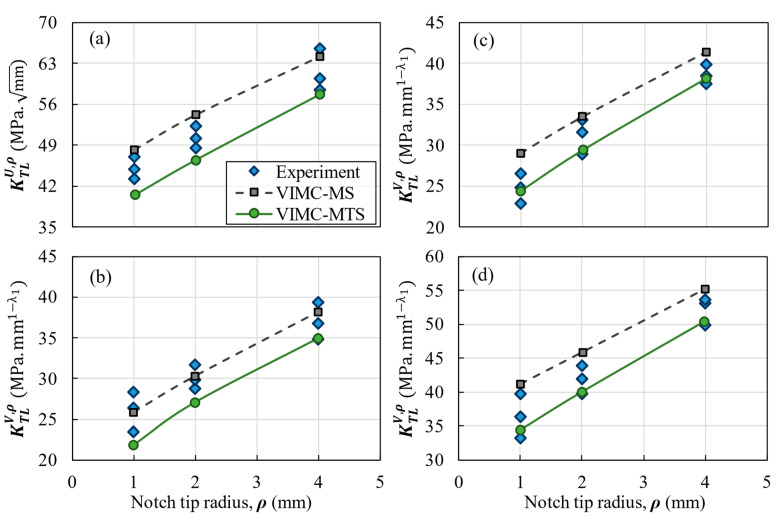
Experimental and predicted TLNFT of the unidirectional laminate versus the notch-tip radius. (**a**) U-notch; (**b**) V-notch with a 30° opening angle; (**c**) V-notch with a 60° opening angle; (**d**) V-notch with a 90° opening angle.

**Figure 8:**
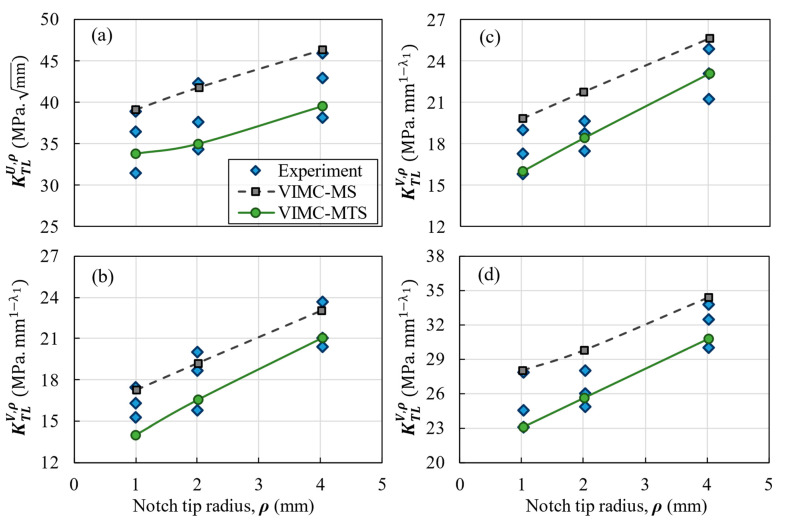
Experimental and predicted TLNFT of the cross-ply laminate versus the notch-tip radius. (**a**) U-notch; (**b**) V-notch with a 30° opening angle; (**c**) V-notch with a 60° opening angle; (**d**) V-notch with a 90° opening angle.

**Figure 9:**
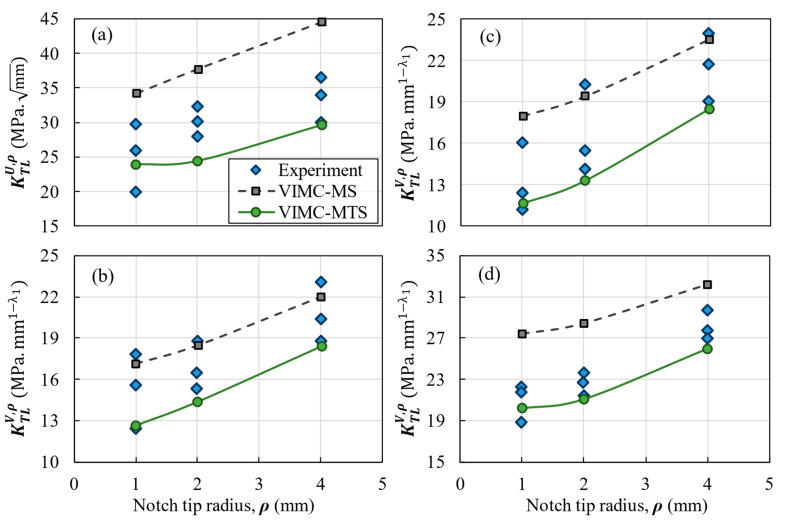
Experimental and predicted TLNFT of the quasi-isotropic laminate versus the notch-tip radius. (**a**) U-notch; (**b**) V-notch with a 30° opening angle; (**c**) V-notch with a 60° opening angle; (**d**) V-notch with a 90° opening angle.

The comparison between the experimental results and the predictions provided by both the VIMC-MTS and the VIMC-MS mixed criteria is shown for each composite configuration in Figure 7, Figure 8 and Figure 9. These figures illustrate the TLNFT (K_TL_^U,ρ^ or K_TL_^V,ρ^) against the notch-tip radius. It can be observed that there was good agreement between the experimental results and the two theoretical criteria for all notch configurations. It can also be deduced that the VIMC-MS mixed criterion always provides more conservative predictions compared to the VIMC-MTS criterion.

Table 5 lists the deviations between the theoretical and the experimental results for all the tests. The results reveal that both criteria were typically successful in predicting the K_TL_^U,ρ^ or K_TL_^V,ρ^ of the notched BD specimens. For example, in the quasi-isotropic lay-up configuration, the average deviations in the VIMC-MTS and VIMC-MS mixed criteria were −6.8% and 8.7%, respectively. The highest deviation in Table 5 is 21.9%, which corresponds to the application of the VIMC-MS mixed criterion to cross-ply laminates with a 90° notch opening angle and a 1 mm notch-tip radius. However, the average deviations of −4.4% for the VIMC-MTS mixed criterion and 11.1% for the VIMC-MS mixed criterion show that both criteria generally provided very good estimations. While the other fracture analysis methods based on microscopic and ply-by-ply approaches in notched laminated composites reported a 7% deviation [44] and a deviation from 1% to 15% [45] between the experimental data and model results, the deviation of the present method in the absence of complicated calculations and time-consuming methods did not look much different from the others. It is also worth mentioning that, in general, the experimental results fall between the VIMC-MTS and the VIMC-MS predictions, with the former providing underestimations of the TLNFT and the latter providing overestimations of this parameter.

## 4. Conclusions

Notched composite Brazilian disk specimens with various notch-tip radii (1 mm, 2 mm, and 4 mm), notch opening angles (0°, 30°, 60°, and 90°), and lay-up configurations (unidirectional, cross-ply, and quasi-isotropic), and subjected to pure mode I loading conditions were analyzed experimentally and theoretically. A total of 18 mechanical characterization tests and 108 mode I notch fracture tests were originally conducted. In order to avoid using complex models for predicting last ply failure loads, the virtual isotropic material concept (VIMC), in conjunction with two brittle fracture criteria (maximum tangential stress, MTS, and mean stress, MS), was applied. The stress field required for calculating the theoretical translaminar fracture toughness of the notched samples was obtained by simulating the samples using the finite element method and assuming an isotropic behavior. Both the VIMC-MTS and VIMC-MS mixed criteria were shown to estimate the experimental findings accurately, as the average difference percentages between theory and experiments were −4.4% and 11.1%, respectively.

## Figures and Tables

**Table 1:** Mechanical properties of the tested laminates (16 layers on each lay-up configuration).

	Unidirectional	Cross-Ply	Quasi-Isotropic
σ_u_ (MPa)	876 ± 4.0	498 ± 4.4	442 ± 5.3
K_TL_ (MPa·m^1/2^)	47.8 ± 1.3	36.5 ± 2.7	40.2 ± 5.3
E (GPa)	46.0	31.1	34.0

**Table 2:** Experimental fracture loads (N) of unidirectional composite Brazilian disks.

Notch Type	Notch Radius (mm), *ρ*	P_1_	P_2_	P_3_	P_average_	Standard Deviation
U-notch	1	6611	6520	6860	6663	176.0
	2	7050	7230	6970	7083	133.2
	4	8100	8430	8764	8431	332.0
V-notch, 30°	1	6530	6320	6070	6306	230.3
	2	6910	7050	6840	6933	106.9
	4	7680	7500	7800	7660	151.0
V-notch, 60°	1	5420	5210	5100	5243	162.6
	2	5870	5900	5770	5846	68.1
	4	6620	6450	6920	6663	238.0
V-notch, 90°	1	4150	4810	3940	4300	454.0
	2	4690	5170	4670	4843	283.1
	4	5730	5450	5920	5700	236.4

**Table 3:** Experimental fracture loads (N) of cross-ply composite Brazilian disks.

Notch Type	Notch Radius (mm), *ρ*	P_1_	P_2_	P_3_	P_average_	Standard Deviation
U-notch	1	5120	5440	6050	5536	472.4
	2	5660	5710	6300	5890	355.9
	4	7711	7673	7260	7548	250.1
V-notch, 30°	1	4520	4550	5100	4723	326.5
	2	6100	5420	5640	5720	347.0
	4	6800	7300	6900	7000	264.6
V-notch, 60°	1	3470	3510	4150	3710	381.6
	2	5040	4790	4530	4786	255.0
	4	5780	6450	5960	6063	346.7
V-notch, 90°	1	3230	3100	3540	3290	226.0
	2	4160	3965	3670	3923	246.7
	4	4970	5310	5100	5126	171.5

**Table 4:** Experimental fracture loads (N) of quasi-isotropic composite Brazilian disks.

Notch Type	Notch radius (mm), *ρ*	P_1_	P_2_	P_3_	P_average_	Standard Deviation
U-notch	1	7900	8300	8430	8210	276.2
	2	8900	8300	8430	8550	315.6
	4	9330	8940	9420	9230	255.1
V-notch, 30°	1	7130	7480	7850	7486	360.0
	2	7300	8200	7800	7766	450.9
	4	8100	8400	8700	8400	300.0
V-notch, 60°	1	6190	6580	6730	6500	278.7
	2	6250	7380	6730	6786	567.1
	4	7060	7530	7670	7420	319.5
V-notch, 90°	1	5260	5780	5960	5666	363.5
	2	5430	6470	5650	5850	548.1
	4	6110	6470	7100	6560	501.1

**Table 5:** Deviations between experimental and theoretical results for the different composite configurations and sample geometries.

Composite Configuration	*ρ* (mm)	Deviation VIMC-MTS Criterion (%)				Deviation VIMC-MS Criterion (%)			
		U-Notch	30°	60°	90°	U-Notch	30°	60°	90°
Unidirectional	1	−6.1	−6.7	−7.3	−4.8	10.6	9.6	9.6	11.9
	2	−7.9	−6.2	−5.3	−3.2	7.1	5.0	6.6	9.2
	4	−4.3	−4.1	−3.9	−4.1	6.4	3.2	3.3	3.9
Cross-ply	1	−8.4	−7.2	−7.3	−0.7	6.8	11.9	11.9	21.9
	2	−7.6	−3.7	−6.2	−2.1	9.4	16.9	9.0	15.2
	4	−3.1	−0.5	−0.4	−5.3	17.3	8.4	10.1	6.4
Quasi-isotropic	1	−7.7	−7.9	−6.1	−6.8	5.1	12.8	13.8	11.8
	2	−10.7	−8.9	−9.9	−5.3	6.0	9.4	8.9	12.5
	4	−5.5	−5.5	−5.3	−7.2	11.0	7.3	8.3	7.3

## Data Availability

The data presented in this study are available on request from the corresponding authors.
